# Territory surveillance and prey management: Wolves keep track of space and time

**DOI:** 10.1002/ece3.3176

**Published:** 2017-09-09

**Authors:** Ulrike E. Schlägel, Evelyn H. Merrill, Mark A. Lewis

**Affiliations:** ^1^ Department of Mathematical and Statistical Sciences University of Alberta Edmonton AB Canada; ^2^ Plant Ecology and Nature Conservation Institute of Biochemistry and Biology University of Potsdam Potsdam Germany; ^3^ Department of Biological Sciences University of Alberta Edmonton AB Canada

**Keywords:** animal movement, cognition, GPS data, landscape of fear, movement ecology, predator–prey, spatial memory, step selection, territoriality, time since last visit

## Abstract

Identifying behavioral mechanisms that underlie observed movement patterns is difficult when animals employ sophisticated cognitive‐based strategies. Such strategies may arise when timing of return visits is important, for instance to allow for resource renewal or territorial patrolling. We fitted spatially explicit random‐walk models to GPS movement data of six wolves (*Canis lupus*; Linnaeus, 1758) from Alberta, Canada to investigate the importance of the following: (1) territorial surveillance likely related to renewal of scent marks along territorial edges, to reduce intraspecific risk among packs, and (2) delay in return to recently hunted areas, which may be related to anti‐predator responses of prey under varying prey densities. The movement models incorporated the spatiotemporal variable “time since last visit,” which acts as a wolf's memory index of its travel history and is integrated into the movement decision along with its position in relation to territory boundaries and information on local prey densities. We used a model selection framework to test hypotheses about the combined importance of these variables in wolf movement strategies. Time‐dependent movement for territory surveillance was supported by all wolf movement tracks. Wolves generally avoided territory edges, but this avoidance was reduced as time since last visit increased. Time‐dependent prey management was weak except in one wolf. This wolf selected locations with longer time since last visit and lower prey density, which led to a longer delay in revisiting high prey density sites. Our study shows that we can use spatially explicit random walks to identify behavioral strategies that merge environmental information and explicit spatiotemporal information on past movements (i.e., “when” and “where”) to make movement decisions. The approach allows us to better understand cognition‐based movement in relation to dynamic environments and resources.

## 1 INTRODUCTION

Recent empirical and theoretic work suggests that cognition and memory are important for animals’ daily movements (Fagan et al., 2013). For example, spatial memory and memory of past experience allow animals to revisit profitable foraging locations and optimize energy intake (Hopkins, 2015; Merkle, Fortin, & Morales, 2014; Nabe‐Nielsen, Tougaard, Teilmann, Lucke, & Forchhammer, 2013; Riotte‐Lambert, Benhamou, & Chamaillé‐Jammes, 2015; Van Moorter et al., 2009) or to travel efficiently to crucial resources such as waterholes (Polansky, Kilian, & Wittemyer, 2015). Cognitive abilities are associated with metabolic needs (e.g., larger brain size, maintenance of neural structures) and may entail both constitutive and induced costs in terms of fecundity and other fitness components (Burns, Foucaud, & Mery, 2011). Therefore, we would expect to find cognitive‐based movement predominantly under conditions where benefits can outweigh costs, that is when resources are heterogeneous in space and time but also predictable (Avgar, Deardon, & Fryxell, 2013; Mueller, Fagan, & Grimm, 2011), and when resource patch density is low and distances between patches are high (Bracis, Gurarie, Van Moorter, & Goodwin, 2015; Grove, 2013). Despite the growing effort in addressing cognition in movement studies and the evidence that it can be important, unraveling the role of cognition and memory for movement is still inherently difficult because these processes can be inferred only indirectly, which requires both creative and state‐of‐the‐art methodology (Fagan et al., 2013).

Here, we address whether gray wolves (*Canis lupus*) integrate spatiotemporal aspects (i.e., the “when” and “where”) of their own travel history into their movement decisions. That memory of travel history is important in wolf movement decisions is reasonable because wolves exhibit little daily overlap in use of their territory, especially in winter, and it raises the questions as to the underlying mechanism (Jedrzejewski, Schmidt, Theuerkauf, Jedrzejewska, & Okarma, 2001). We use a novel method of modeling memory‐based animal movements (Schlägel & Lewis, 2014) to assess hypotheses (Table 1) related to the role of time‐dependent territorial and hunting behavior based on time since last visiting (TSLV) a location.

**Table 1:** Tested hypotheses regarding drivers of wolf movement. Our main interest lies in testing time‐dependent movement strategies (H_2_, H_5_, and H_6_) but we included time‐independent movement behaviors as possible simpler explanations (H_0_, H_1_, H_3_, and H_4_). The probability of selecting a location is modeled as a logistic weighting function of the spatial attributes time since last visit (TSLV), distance from territory edge (edge), and prey density (prey) within a spatially explicit movement model. For hypotheses involving two spatial attributes, we tested both a model with additive term in the linear predictor (resulting in a shift of the logistic weighting function) and a model with additional multiplicative interaction (changing also the steepness of the logistic weighting function)

Hypothesis	Behavior		Spatial attributes (model)	Expected relationship with probability of selection			
				TSLV	Distance from edge^a^	Prey density	Interaction
No preferences	General movement tendencies only	H_0_	– (null)	–	–	–	–
Risk avoidance	Avoidance of territory edge	H_1_	Edge	–	Pos	–	–
Territory surveillance	Avoidance of edge, but reduced for long TSLV	H_2a_	TSLV + edge	Pos	Pos	–	
		H_2b_	TSLV + edge + TSLV × edge	Pos^b^	Pos^b^	–	Pos
Prey selection	Preference for high prey density	H_3_	Prey	–	–	Pos	–
Prey selection & risk avoidance	Preference for high prey density but avoid edge	H_4a_	Edge + prey	–	Pos	Pos	–
		H_4b_	Edge + prey + edge × prey	–	Pos^b^	Pos^b^	Pos
Delayed return	Preference for long TSLV	H_5_	TSLV	Pos	–	–	
Prey management	Preference for long TSLV; high prey density induces earlier return	H_6a_	TSLV + prey	Pos	–	Pos	–
		H_6b_	TSLV + prey + TSLV × prey	Pos^b^	–	Pos^b^	Pos

a A positive coefficient for this attribute means that the probability of selecting a location increases with its distance from the edge, that is, toward central locations.

b Dominant effect of the attribute on the probability of selection (a negative coefficient can be compensated for a range of attribute values by a positive interaction term).

Wolves are known to be territorial and to scent mark their territories to advertise their presence to wolves from other packs (Lewis & Murray, 1993; Peters & Mech, 1975; Zub et al., 2003). Scent marks can be found across the territory, but usually territory edges are marked more heavily, especially when they border neighboring packs (Mech & Boitani, 2006; Peters & Mech, 1975; Zub et al., 2003). If fatal encounters with individuals from other packs occur close to the territory edge (Mech, 1994), we would expect avoidance of territory edges to be a major driver to wolf movement (risk avoidance; H_1_). However, if scent marks decay and must be renewed regularly, we would expect avoidance of territory edges to decline for long TSLV (territory surveillance; H_2a_, H_2b_) due to renewing scent marks.

Movement of wolves also may be driven by strategies for efficient prey capture. For example, selecting areas of high prey density (prey selection; H_3_) would reduce search time to find and potentially kill a prey (Holling, 1959; McPhee, Webb, & Merrill, 2012). However, if prey concentrate in buffer zones between wolf territories that act as refuges to prey (Mech, 1994), wolves are faced with making trade‐offs in finding prey while at the same time avoiding conspecifics from other packs (prey selection and risk avoidance; H_4a_, H_4b_).

Prey can exhibit temporary avoidance, heightened vigilance, or retreat to safer habitats in areas of recent wolf presence or where conspecifics were recently killed by wolves (Berger‐Tal & Bar‐David, 2015; Latombe, Fortin, & Parrott, 2014; Liley & Creel, 2008). Contrary to predictions by the “risky places hypothesis,” which accounts only for varying antipredator behavior across sites with different long‐term predation risk, observations of elk responses to wolves suggest that antipredator behavior adjusts dynamically to the presence of wolves in line with the “risky times hypothesis” and the “risk allocation hypothesis” (Creel, Winnie, Christianson, & Liley, 2008; Robinson & Merrill, 2013). These behavioral responses lower predation success, an effect called behavioral depression of prey (Charnov, Orians, & Hyatt, 1976). To optimize hunting success, wolves may not only optimize giving‐up times (Brown, Laundré, & Gurung, 1999; Charnov et al., 1976), but also select for longer TSLV (delayed return; H_5_) to allow time for prey behavior to recover (Latombe et al., 2014; Laundré, 2010). This also spreads the risk over all hunting sites (Lima, 2002). However, wolves may return sooner to areas of high prey density (prey management; H_6a_, H_6b_) because of success in finding prey (Kunkel & Pletscher, 2001; McPhee et al., 2012) and greater variation in recovery times of individual prey.

We examined the support for these hypotheses in a model selection framework using movement data of six GPS‐collared wolves in winter when denning is less likely to influence movement, and packs are likely to be more cohesive (Metz, Vucetich, Smith, Stahler, & Peterson, 2011). We contrasted our behaviorally based models with a null model that assumed no preferences for spatiotemporal behaviors (H_0_). We fit observed movement trajectories to random walks that included behavioral mechanisms via a spatially explicit and dynamic resource‐selection component (Schlägel & Lewis, 2014). With this, we illustrate how to detect an interplay of travel history with current movement decisions in movement patterns of free‐ranging animals.

## 2 MATERIALS AND METHODS

### 2.1 Wolf and ungulate prey data

Data were collected during 2004–2009 in a 25,000 km^2^ area west of Rocky Mountain House, Alberta, Canada (52°27′N, 115°45′W). The area is part of the central east slopes of the Rocky Mountains, and terrain includes gentle foothills in the eastern parts as well as mountains (<3,100 m) toward the west. Much of the landscape is covered by conifer forest (52%), which is interspersed with smaller areas of natural lowlands (10%), forestry cut‐blocks (6%), stands of deciduous forest (3%) with the remaining being largely permanent ice and rock (Webb, Hebblewhite, & Merrill, 2008).

During the years 2004–2006, wolves were captured and fitted with GPS collars (Lotek 3300Sw and 4400S; for details, see Webb et al., 2008). The collars were programmed to collect location measurements every 2 hr. This led to regular time series of observed movement steps. Successful fix attempts for locations were 90% (3300Sw model) and 82% (4400S model) indicating habitat‐induced GPS bias was minimal (Frair et al., 2004; Hebblewhite, Percy, & Merrill, 2007). We analyzed data of six wolves from different packs whose territories were in the eastern part of the study area with low elevation and no mountain valleys. The movement data of the six wolves used in the analysis started between 3 November and 2 January and spanned until 23 February and 14 April, depending on individual, spanning on average 121 days (SD 23) and with an average of 1,458 (SD 289) locations/wolf.

The five major ungulate prey species for wolves were white‐tailed deer (*Odocoileus virginiana*), mule deer (*O. hemionus*), elk (*Cervus elaphus*), moose (*Alces alces*), and feral horses (*Equus caballus*) and comprised 92–96% of the prey biomass within wolf scat (Merrill, unpublished data). To obtain spatially explicit maps of densities, fecal pellet groups deposited over winter were counted across 372 transects (1 km × 2 m) after snow melt. Pellet counts from transects were interpolated across the study area using inverse‐distance weighting. Counts of pellet groups were converted to individual numbers of elk and moose based on ratios of number of pellet groups to the estimated number of individuals within 16 wildlife management units obtained through winter aerial surveys. For deer and feral horses, there were no aerial surveys so the ratio obtained for moose was adjusted for deer and horses based on differences in winter defecation rates of the species (McPhee et al., 2012).

To obtain a combined measure of available prey density for all four species, we calculated a weighted sum of all prey numbers, where weights were based on average ungulate body mass in winter (Knopff, Knopff, Kortello, & Boyce, 2010; see Appendix A1). Prey densities (number/30 m^2^) were aggregated to a spatial resolution of 300 m × 300 m cells, mainly for computational limitations (see benchmarks in Appendix A3); however, wolves likely can detect prey within this distance (Basille et al., 2015; Kuijper et al., 2014). Wolf movement trajectories were considered accordingly on this spatial grid of cells, using the coordinates of the cell centers. Each location of a wolf was attributed to the grid cell in which it fell.

### 2.2 Spatial information and travel history

Relocation data were analyzed using statistical movement models developed by Schlägel and Lewis (2014). These models are spatially explicit random walks in which spatial information influences movement decisions. The random walk is performed on a discrete grid of cells in correspondence to the prey density data. To test the hypothesized explanations of wolf movement behavior (Table 1), three types of spatial attributes were considered. First, the combined prey density measure (prey) was normalized over the territory (see next paragraph) of each wolf. Second, for each territory, the minimum distance of each location from the territory edge (edge) was calculated. Distance from edge is zero at the territory edge and increases for locations more centered within the territory. Third, time since last visit (TSLV) was based on an individual's own travel history. TSLV was defined to specify at each time step, and for each location, the time (measured in time steps) since the animal had last been to the location, that is, grid cell. TSLV is a dynamic attribute of a grid cell that changes according to the individual's movement. TSLV increases for locations that the individual stays away from and is reset to 1 whenever the individual visits a location. Locations were considered visited when they lay within a buffer zone of the straight‐line path between two locations. The buffer zone included four grid cells, corresponding to approximately 1,200 m (see Appendix A1 for an explanation and justification). To initialize TSLV, we started the wolf at the first telemetry location and used an initial phase of 300 time steps, representing 25 days, which was excluded from further analysis. Before inclusion in the weighting function, all TSLV values were log‐transformed because values had a wide range across the territory, with few very large values. For further information on TSLV, see Appendix A1.

**Figure 1:**
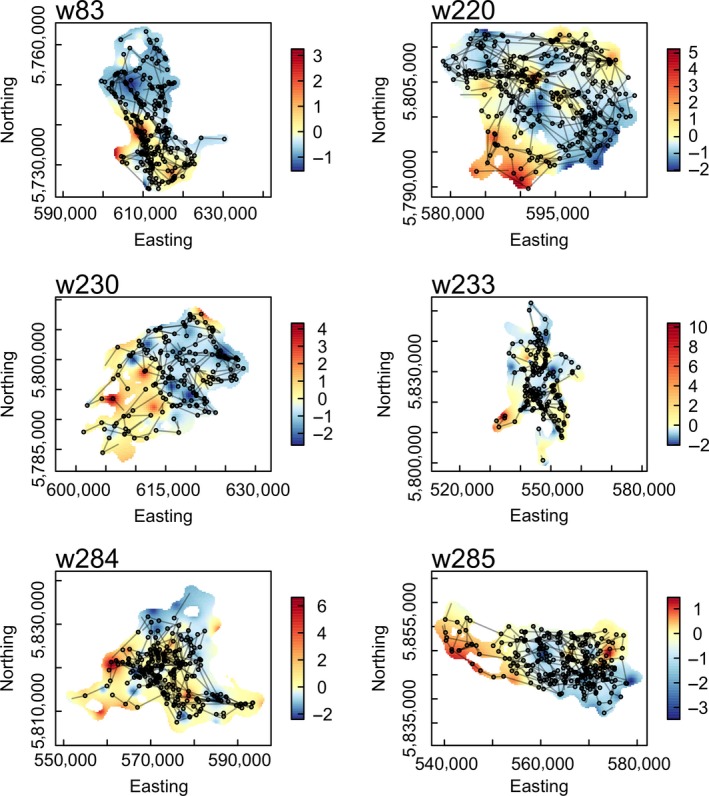
Maps of winter movements of six individual wolves during 10–20 weeks. Colors reflect standardized prey density. Prey density is a combined measure of densities of the main ungulate prey species (deer, elk, moose, feral horse). Black circles are wolf locations with black lines indicating the straight‐line steps between locations. Depicted are only “relocating” steps used for the anysis and exclude non‐relocating steps such as when handling prey and resting (number of relocating steps was 177–332)

A territory was defined for each wolf based on a Brownian bridge kernel estimate of the individual's utilization distribution obtained with R package “adehabitatHR” (Calenge, 2006; Horne, Garton, Krone, & Lewis, 2007). For this estimation, we used all locations including the first 300 steps for initializing TSLV. The purpose of the territory was twofold. We used it to estimate the “edge” of the territory, close to which the mortality risk due to aggressive encounters with other wolf packs may be higher. We also used the edge as a reflective boundary in the movement model to avoid an artificial avoidance of areas with long TSLV that were not visited during our study period for possibly external reasons (e.g., other pack activity). Therefore, the territory included all locations within the 99.9% quantile of the estimated utilization distribution (Figure 1), which was the area that contained all locations possibly relevant for an individual during the study period.

### 2.3 Movement model

In the models, two aspects affect the probability for a movement step between times *t *− 1 and *t* from location $x_{t-1}$ to *x*_*t*_. First, a movement kernel *k* describes general tendencies regarding speed and directional persistence. Here, the kernel is composed of a Weibull distribution for step lengths and a uniform distribution for bearings (Appendix A1). Second, given a probability distribution for a step based on the kernel *k*, a weighting function *w* adjusts these probabilities based on preferences for the three spatial attributes, which are encoded in the vector $F_{t}$. Because the model is spatially explicit, each location *x* has its own values of the spatial attributes, that is $F_{t}\left(x\right)=\left(\text{prey}\left(x\right),\text{edge}\left(x\right),\text{TSLV}\left(x\right)\right)$. The overall step choice probability is given by

(1) $$p\left(x_{t}|x_{t-1}\right)=\frac{k\left(x_{t};x_{t-1}\right)w\left(F_{t}\left(x_{t}\right)\right)}{\sum_{z\in \Omega }k\left(z;x_{t-1}\right)w\left(F_{t}\left(z\right)\right))}.$$

We recall that locations represent discrete 300 × 300‐m cells in space. The sum in the denominator is a normalization constant over a large enough area Ω around the current location such that the probability of stepping outside this area is negligible. The radius of the area Ω (ranging 30–44 cells, i.e., 9.0–13.2 km) was chosen to be larger than the longest step taken by the wolf. Steps outside the territory have probability zero.

The weighting function is modeled after a resource selection probability function (Lele, Merrill, Keim, & Boyce, 2013), giving the binomial probability of selecting a location *x* based on the attributes of the location, $F_{t}\left(x\right)$. Here, we used a logistic form,

(2) $$w\left(F_{t}\left(x\right)\right)=\frac{1}{1+e^{-f(F_{t}\left(x\right),\;\alpha ,\;\beta ,\;\gamma )}}$$

The predictor term $f(F_{t}\left(x\right),\;\alpha ,\;\beta ,\;\gamma )$ contains additive and multiplicative combinations of the spatial attributes, according to our hypotheses (Table 1). For the no preference model, the weighting function is constant over space, that is, $f(F_{t}\left(x\right),\;\alpha ,\;\beta ,\;\gamma )=0$, and only the kernel *k* influences movement. In the models risk avoidance, prey selection, and delayed return, the weighting function includes one spatial attribute, and the predictor term $f(F_{t}\left(x\right),\alpha ,\beta ,\gamma )$ is simply given by $\alpha +\beta_{\mathrm{tslv}}\cdot {\text{TSLV}}_{t}\left(x\right),\;\alpha +\beta_{\mathrm{edge}}\cdot \text{edge}\left(x\right),$ or $\alpha +\beta_{\mathrm{prey}}\cdot \text{prey}\left(x\right)$, for each model, respectively. The parameter α is the intercept of the predictor term. For a sigmoidal logistic function, it determines the position of the inflection point of the curve, that is, where the function reaches the value 0.5. For hypotheses that involved two spatial attributes, two models were considered, one with additive term only and one with additional multiplicative interaction. For the model prey selection and risk avoidance, the additive term is $\alpha +\beta_{\mathrm{edge}}\cdot \text{edge}\left(x\right)+\beta_{\mathrm{prey}}\cdot \text{prey}\left(x\right)$ (H_4a_, H_4b_) and the multiplicative term is $\gamma_{e,p}\cdot \text{edge}\left(x\right)\cdot \text{prey}\left(x\right)$ (H_4b_). The models territory surveillance and prey management were built analogously, with interaction parameters $\gamma_{t,e}$ and $\gamma_{t,p}$, respectively. The parameters α, $\beta_{\mathrm{tslv}}$, $\beta_{\mathrm{edge}}$, $\beta_{\mathrm{prey}}$, $\gamma_{e,p}$, $\gamma_{t,e}$, $\gamma_{t,p}$ determine the direction and strength of preferences.

Following Aarts, Fieberg, and Matthiopoulos (2012), the weighting function $w(F_{t}\left(x\right))$ is a function of geographical space, *x*, via the spatial attributes $F_{t}\left(x\right)$ at a location *x*. It can alternatively be viewed as a weighting function over environmental space, F, where attribute values F range over the three different spatial attributes TSLV, prey, and edge. This latter perspective allows an interpretation of the effects of spatial attributes on movement decisions similar to a classical step‐selection analysis (Fortin et al., 2005). When considering the weighting function in environmental space, w(F), as a function of one variable, for example, *w*(TSLV), it is a sigmoidal curve. Additional additive terms of the other attributes (having β coefficients) shift the curve, whereas multiplicative terms (having γ coefficients) additionally influence the nonlinearity or shape of the curve. A shift in the curve means that the switch from an avoidance (small probability of selection) to a preference (high probability of selection) of a location happens at a different value of the spatial attribute. If the steepness of the curve increases (decreases), the switch happens more (less) abruptly.

### 2.4 Statistical analysis

Movement data were analyzed individually for each wolf, comparing the fit of 10 models (Table 1). Wolves express different behavioral modes, such as handling a kill, resting away from a kill site, or relocating to a new location (Franke, Caelli, Kuzyk, & Hudson, 2006; Merrill et al., 2010). Because our goal was to understand the effect of TSLV with respect to the territory surveillance and revisiting areas of varying prey densities, we used only relocating movement steps for model fitting. Relocating steps were considered those that spanned at least five cells (1,500 m) in our discretized space (Franke et al., 2006; see Appendix A1 for details). However, non‐relocating steps were omitted only after calculating TSLV for the entire time series ensuring appropriate values of TSLV that represented the correct times based on the full path. The final data comprised 244, 322, 181, 177, 251, and 276 steps for individuals w83, w220, w230, w233, w284, and w285, respectively.

Maximum‐likelihood estimates of the model parameters were obtained by numerically optimizing the model's likelihood function using a Nelder–Mead algorithm implemented in R (R Core Team 2015, Appendix A1). Model selection was performed via Akaike information criterion (AIC). We used the small sample criterion AIC_c_ because ratios of available steps to number of parameters for the most complex model were ≤40 (Burnham & Anderson, 2002). Within nested models, more complex models were selected when AIC_c_ differences were larger than 2. We based this on the rule of thumb given by Burnham and Anderson (2002) that AIC differences of 2 or smaller indicate substantial support for a model. Adhering to the principle of parsimony, we therefore only selected a more complex model when its ΔAIC_c_ was larger than 2 compared to the next simpler model. Parameter estimates of the weighting function were analyzed for their effects on movement decisions using the representation of the weighting function in environmental space, w(F) (Aarts et al., 2012).

## 3 RESULTS

### 3.1 General movement tendencies

Based on the best‐fit model, mean displacements over 2‐hr time intervals (relocating steps only), calculated from parameters of the Weibull distribution for step lengths in the movement kernel *k*, ranged from 2,500 to 3,600 m (±300 m due to the spatial discretization) for the six wolves (Table 2). When comparing this with estimates based on the null model, there is a consistent trend. Estimates of both the shape (*λ*) and scale (σ) of the Weibull distribution were smaller for the best‐fit model, which included selection for spatial attributes, than for the null model (Table 2). The null model distribution corresponds to the “empirical kernel” used in classic step‐selection analyses to sample “control” steps (Fortin et al., 2005). Here, this would have consistently overestimated step length by approximately 300–570 m per 2‐hr step.

**Table 2:** Parameter estimates, together with standard errors (*SE*) of the kernel *k* describing general movement tendencies (i.e., step length). The parameters are the shape (*λ*) and scale (σ) of the Weibull distribution used to model step length. The last column gives the mean of the resulting Weibull distribution. For each wolf, we show the parameter estimates from the best model compared to the null model. The null model consistently overestimates general tendencies for step length

	*λ*	*SE* (*λ*)	σ	*SE* (σ)	Mean^a^
w83					
Null	2.45	0.12	14.39	0.42	12.76
Best	2.04	0.14	13.24	0.14	11.73
w200					
Null	2.23	0.09	12.94	0.36	11.46
Best	1.82	0.10	11.41	0.10	10.14
w230					
Null	2.62	0.14	10.97	0.34	9.75
Best (edge)	2.02	0.17	9.47	0.17	8.39
Best (prey)	2.04	0.17	9.50	0.17	8.41
w233					
Null	2.20	0.12	13.19	0.50	11.68
Best	1.77	0.14	11.46	0.14	10.20
w284					
Null	1.90	0.08	15.61	0.59	13.86
Best	1.47	0.45	13.21	0.45	11.95
w285					
Null	2.21	0.09	12.52	0.38	11.09
Best	1.84	0.27	11.17	0.27	9.92

a Because the analysis operated on 300 × 300 m cells, the mean values translate into meters via multiplication by 300, for example, a mean of 10 translates into a mean step length of 3 km ± 300 m.

### 3.2 Selection for spatial attributes

For all six wolves, the territory surveillance model with interaction of TSLV and distance from territory edge (H_2b_) had minimum AIC_c_ (Table 3). For one individual, w230, the same minimum AIC_c_ was reached by the prey management model with additive terms of TSLV and prey (H_6a_). The territory surveillance and prey management hypotheses are not mutually exclusive, and therefore both could be supported by the data without contradiction. Because this suggested the importance of both territory surveillance and prey management, we also tested a combined model with these terms (TSLV + edge +prey + TSLV × edge + TSLV × prey) in the weighting function. For w230, this became the best model, and for w284 the model performed similarly well as the territory surveillance model but was neither significantly better nor parsimonious (Table A1 in Appendix A2).

**Table 3:** Model selection results for the six wolves. Presented are AIC_c_ differences, ΔAIC_c,i_ = AIC_c,i_ – AIC_c,min_ for each model i. Best models are highlighted in bold. For all individuals, the best model includes TSLV and distance from territory edge with multiplicative interaction, supporting the territory surveillance hypothesis. For individual w230, the first rank is shared with the model that includes additive terms of TSLV and prey density, supporting the prey management hypothesis

		ΔAIC_c_					
		w83	w220	w230	w233	w284	w285
H_0_	Null	59.7	66.4	57.9	63.4	125.9	58.0
H_1_	Edge	45.2	66.6	55.6	49.7	76.6	38.2
H_2a_	TSLV + edge	17.6	6.5	7.9	27.7	53.4	23.6
H_2b_	**TSLV** **+ edge + TSLV** × **edge**	**0**	**0**	**0**	**0**	**0**	**0**
H_3_	Prey	63.0	60.7	57.8	67.3	129.9	59.0
H_4a_	Edge + prey	45.6	67.7	44.7	43.3	72.5	33.1
H_4b_	Edge + prey + edge × prey	47.7	69.6	43.5	41.6	74.3	35.1
H_5_	TSLV	16.1	5.8	5.8	36.3	51.8	22.7
H_6a_	**TSLV** + **prey**	18.0	5.9	**0**	30.2	52.7	23.2
H_6b_	TSLV + prey + TSLV × prey	18.0	6.0	2.1	32.0	53.7	22.7

**Figure 2:**
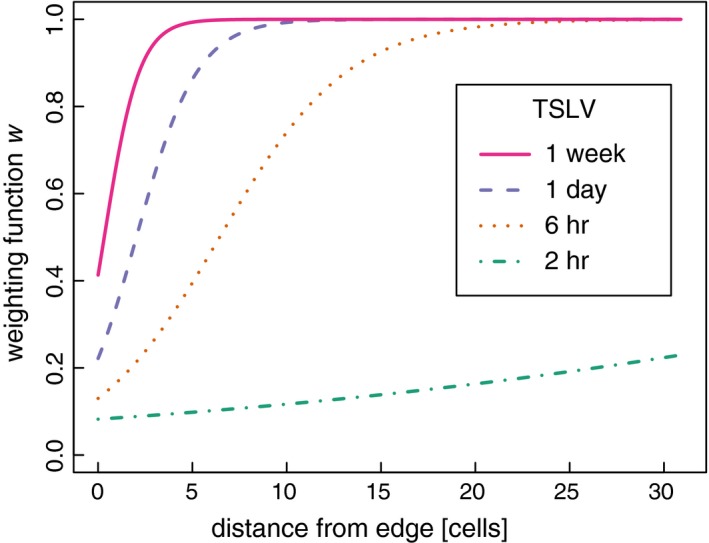
Weighting function for the territory surveillance model (H_2b_) based on parameter estimates from individual w220. This model was best for all wolves. The weighting function gives the probability of selecting a location based on spatial attributes, here depicted as function of distance from territory edge (edge) in environmental space where distance from the edge was measured in discrete cells (300 × 300 m) for varying values of time since last visit (TSLV). The increasing sigmoidal curve indicates that locations closer to the edge are avoided. With increasing TSLV, the curve is shifted to the left due to the positive coefficient ($\beta_{\mathrm{tslv}}$) and becomes steeper due to the positive interaction parameter ($\gamma_{t,e}$), indicating that the avoidance of edge locations vanishes. Graphs for the other individuals show similar patterns Fig. A2

**Figure A2:**
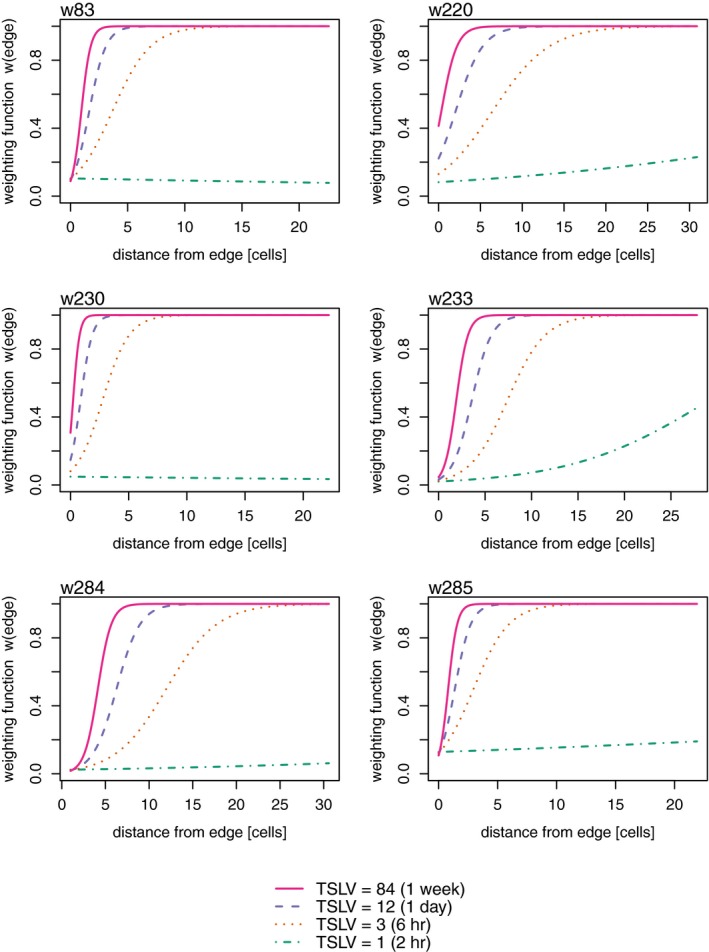
Weighting function for the territory surveillance model (H_2b_) with minimum AIC_c_ using the estimated parameters. Panels correspond to individual wolves. The weighting function gives the probability of selecting a location based on spatial attributes, here depicted as function of distance from territory edge (in environmental space). Distance from the edge is measured in discrete cells (300 × 300 m). For all wolves, the increasing direction of the sigmoidal curve indicates that locations closer to the edge are avoided. With increasing time since last visit (TSLV), the curve is shifted to the left due to the positive coefficient **(** ***β***_tslv_) and becomes steeper due to the positive interaction parameter (***γ***_*t,e*_), indicating that the avoidance of edge locations decreases. The weighting functions for the different wolves show the same general pattern and only vary slightly. This variation is likely due to individual variation of the wolves’ behavior and territories (see also Fig. 1).

Parameter estimates of the weighting function for the territory surveillance model (H_2b_) of all wolves were consistent with our predictions. All multiplicative coefficients ($\gamma_{t,e}$) were positive and their confidence intervals did not overlap zero, while most of the additive coefficients ($\beta_{\mathrm{edge}},\;\beta_{\mathrm{tslv}}$) had confidence intervals that overlapped zero (Table 4). The overall effect of TSLV and edge on the probability of selection (modeled by the weighting function) was dominated by the multiplicative coefficient $\gamma_{t,e}$ and was therefore positive. The overall selection coefficient for edge, given TSLV, was $\beta_{\mathrm{edge}}+\gamma_{t,e}\text{log}\left(\text{TSLV}\right)$. As TSLV increased, this became positive already at TSLV = 2 (4 hr) in all cases. Similarly, the overall selection coefficient for TSLV, given edge, was $\beta_{\mathrm{tslv}}+\gamma_{t,e}\cdot \text{edge}$. As edge increased, starting from 0, this became positive at edge = 1 or 2 (corresponding to approximately 300–900 m from the edge) in all cases. As a result, there was strong evidence for wolves avoiding territorial boundaries, and as TSLV increased, wolf avoidance of the edge declined (Figures 2 and A2). When locations had not been visited for more than approximately 7 days, the weighting function approached a function nearly constant at one, which means that edge and central locations were selected with the same probability.

**Table 4:** Parameter estimates (Est.) and standard error (*SE*) of the best‐fit logistic weighting function *w* based on spatial attributes TSLV, distance from territory edge (edge), and prey density (prey). Parameters β are selection coefficients of additive terms (shifting *w*), and parameters γ describe the multiplicative interaction of two attributes (changing shape of *w* nonlinearly). Parameter α is the intercept of the linear predictor and determines the position of the inflection point of the logistic weighting function where it reaches 0.5. Two best model estimates are given for wolf 230 because they had equal support. Estimates for which Wald‐type 95% confidence intervals do not overlap zero are highlighted in italics

	α	β_tslv_	β_edge_	β_prey_	$\gamma_{e,p}$	$\gamma_{t,e}$	$\gamma_{t,p}$
Territorial surveillance: TSLV + edge + TSLV × edge							
w83							
Est.	*−2.15*	*−*0.043^a^	*−*0.014^a^	–	–	*0.56*	–
*SE*	0.86	0.20	0.091	–	–	0.19	–
w220							
Est.	*−2.41*	*0.47*	0.039	–	–	*0.23*	–
*SE*	0.53	0.15	0.042	–	–	0.082	–
w230							
Est.	*−2.97*	*0.49*	*−*0.016^a^	–	–	*0.82*	–
*SE*	0.80	0.18	0.11	–	–	0.31	–
w233							
Est.	*−3.88*	0.19	*0.13*	–	–	*0.33*	–
*SE*	0.74	0.15	0.054	–	–	0.10	–
w284							
Est.	*−3.74*	*−*0.36^a^	0.033	–	–	*0.28*	–
*SE*	0.82	0.23	0.053	–	–	0.06	–
w285							
Est.	*−1.92*	*−*0.045^a^	0.021	–	–	*0.58*	–
*SE*	0.59	0.18	0.056	–	–	0.22	–
Prey management: TSLV + prey							
w230							
Est.	*−3.96*	*2.66*	–	*−1.54*	–	–	–
*SE*	0.96	0.98	–	0.67	–	–	–

a Negative coefficients for the additive term still resulted in a mostly positive relationship due to the positive interaction coefficient because the overall selection coefficient for TSLV, given edge, is $\beta_{\mathrm{tslv}}\;+\;\gamma_{t,e}\cdot \text{edge}$. Vice versa, the overall selection coefficient for edge, given TSLV, is $\beta_{\mathrm{edge}}\;+\;\gamma_{t,e}\cdot \text{TSLV}$.

**Figure 3:**
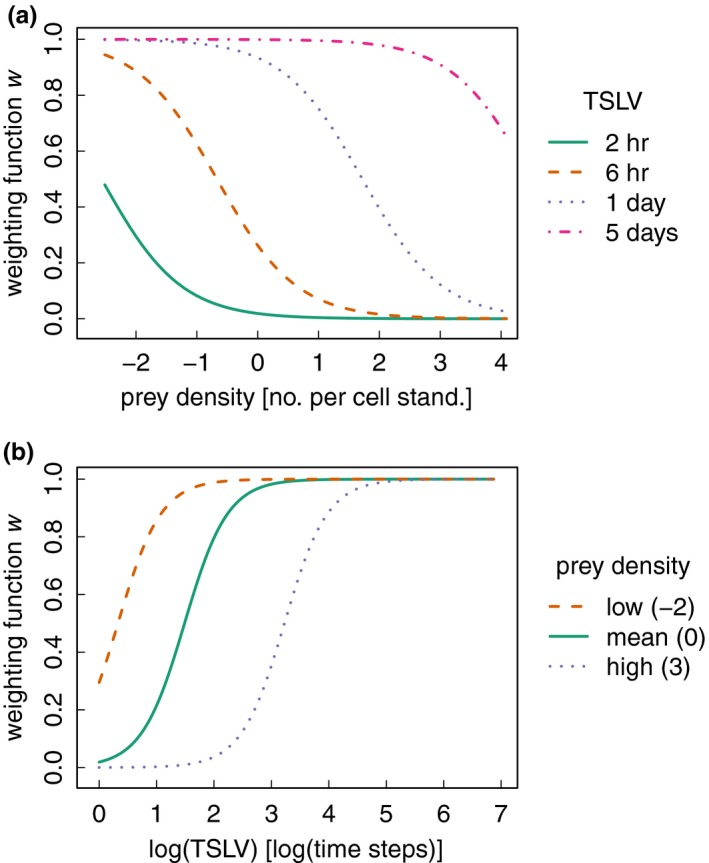
Weighting function for the prey management model (H_6a_), with parameter estimates from individual w230, for which this model shared first rank. The weighting function gives the probability of selecting a location based on spatial attributes, here depicted as function (in environmental space) of **(a**) prey density and **(b**) time since last visit (TSLV). Prey density (numbers per cell) was standardized across the territory. When prey density was high, the probability of selecting a location was higher when time since last visit was longer. When prey density was low, time since last visit mattered less

Movement patterns of wolf w230 also supported the prey management model (H_6a_), where parameter estimates and the resulting weighting function only partly agreed with our expectations in relation to the prey management hypothesis (Table 4). Consistent with our prediction, the selection coefficient $\beta_{\mathrm{tslv}}$ was positive, and therefore the wolf selected for longer TSLV, indicating that returns to previously visited locations were delayed (Table 4, Figure 3b). However, the coefficient β_edge_ was negative and the wolf selected for locations with lower prey density (Table 4, Figure 3a). As a result, the inflection point of the sigmoidal curve from low selection of recently visited sites to high selection of sites with longer absence was shifted to a higher value of TSLV, which led to a longer delay in revisiting sites when prey density was high (Figure 3b). Likewise, the selection for lower prey density was shifted to the right for increasing values of TSLV, which resulted in nearly equal selection for all prey densities after 5 days of absence (Figure 3a).

**Figure A3:**
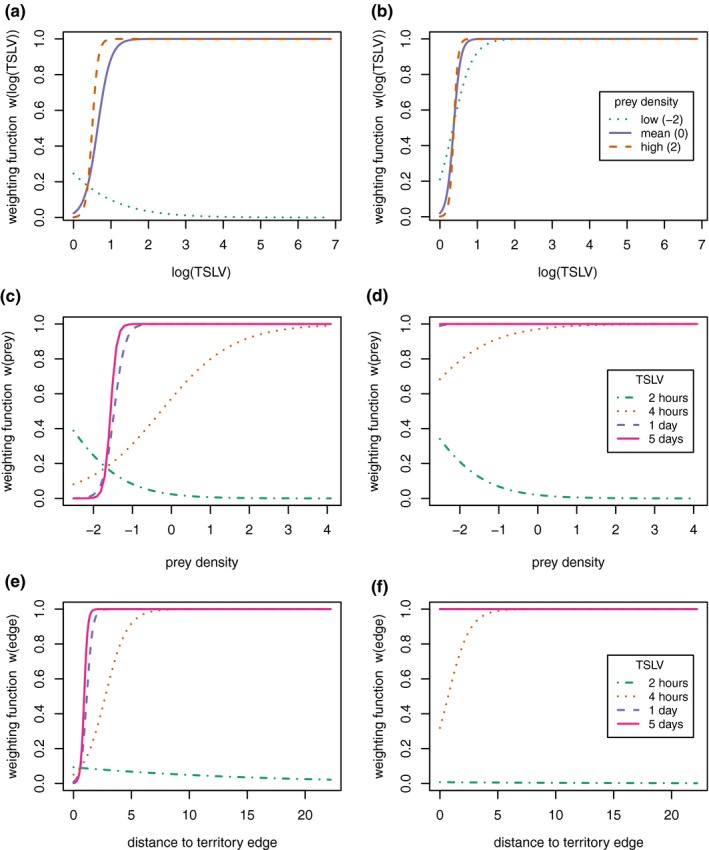
Weighting function for the joint model TSLV+edge+prey+TSLV×edge+TSLV×prey with parameter estimates from individual w230. The weighting function gives the probability of selecting a location based on spatial attributes, here depicted as function in environmental space of the three spatial attributes time since last visit (TSLV) (a and b), prey density (c and d), and distance from territory edge (e and f). TSLV is measured in time steps; prey density (number per cell) is standardized over the territory; distance from edge is measured in cells (300 × 300 m). a and c: Distance from edge is fixed at 2 (approx. 600–900 m from edge). b and d: Distance is fixed at 5 (approx. 1.8–2.1 km from edge). e and f: Prey density is fixed at −1 (below average) and 1 (above average), respectively. The weighting function was nearly constant at 1 across most of the ranges of spatial attributes, with only two notable exceptions. First, abrupt declines to zero selection occurred for locations that had been visited during the last 2‐h‐time step (all panels), which simply may indicate persistent movement. Second, the estimates predict a decline to zero selection for very low prey density close to the edge (a and c), but this effect vanished already slightly further inside the territory (b and d). Thus, effects were predicted only at the most extreme ends of the attribute values. Furthermore, standard errors of parameter estimates were large such that Wald‐type confidence intervals of the parameters would overlap zero (Table A2).

When considering the combined territory surveillance and prey management model for wolf w230, all estimated selection coefficients (all β and γ coefficients) had large confidence intervals that overlapped zero (Table A2). When we plotted the weighting function based on these estimates nonetheless, it was constant at one over most of the range of the spatial attributes, with only two exceptions (Figure A3). First, abrupt declines to zero selection occurred for locations that had been visited during the last 2‐hr‐time step, which simply may indicate persistent movement. Second, the estimates predicted a decline to zero selection for the lowest prey density very close to the edge, and this effect vanished already slightly further inside the territory. Considering also the large and zero‐overlapping confidence intervals, these effects may be over‐fits to spurious effects at the most extreme ends of the attribute values.

## 4 DISCUSSION

We investigated how the time since last visiting a location influenced movement decisions in relation to territory surveillance and prey management. Our models are statistical in the sense that they define a probability distribution for observed movements but mechanistic in that they describe a behavioral movement process. This is in contrast to classic resource (or step) selection analyses that treat movement steps as independent data points and sample control locations (or steps) before estimating selection coefficients (Forester, Im, & Rathouz, 2009; Fortin et al., 2005). The advantage of our method is that parameters of general movement tendencies and spatially explicit preferences are estimated simultaneously without assuming that the two aspects are independent (see also Avgar, Potts, Lewis, & Boyce, 2016), which produces consistently lower estimates of the step length distribution than if independent, a priori estimates for step length would have been used. An additional advantage to this approach that we did not use in this analysis is incorporating directional autocorrelation of movement in the movement kernel *k* (Schlägel & Lewis, 2014). In our case, we did not use this approach because our time series spanned only several weeks, and because we eliminated non‐relocating behaviors such as handling a kill, resting away from a kill site, or revisiting kill sites (Franke et al., 2006; Merrill et al., 2010). Using autocorrelated bearings would have decreased the number of steps available for the analysis even further, because more than two successive location measurements would have been needed to define the probability of a step.

Adjusting returns after previous visitation is important when time is required to replenish high food abundance or quality (Bar‐David et al., 2009; Davies & Houston, 1981; Janmaat, Byrne, & Zuberbühler, 2006; Van Moorter et al., 2009). We found support for an additional type of resource depletion that we hypothesize is related to decay of scent markings. First, there was a general tendency of wolves to avoid locations close to the territory edge, which has been reported elsewhere as a means to elude intraspecific interference along the edge of their territories (Carbyn, 1983; Mech & Harper, 2002). Second, the probability of wolves revisiting these areas increased in time suggesting wolves were responding to a decay in scent marks, which are needed for territorial maintenance (Peters & Mech, 1975; Zub et al., 2003). Scent marks contain pheromones and chemical signals that elicit responses from other individuals and can prevent direct, aggressive encounters (Mech, 1994). They are thought to be an effective means of advertisement because the scent remains in the environment for some time and is readily detected even at night (Feldhamer, Drickamer, Vessey, & Merritt, 2004). Peterson (1974) found on Isle Royale that wolves reversed direction of travel and retreated when they encountered a foreign scent mark along the edge of their territory. Ausband, Mitchell, Bassing, and White (2013) also reported that wolves will avoid areas where humans place wolf scats if they are regularly maintained. Indeed, the consistency in territorial surveillance among all six wolves indicates there is strong motivation for rotational movements to revisit the territory edge for territory maintenance (Jedrzejewski et al., 2001).

In contrast, we found less support for prey density influencing wolf movements and for movements being consistent with behavioral depression of prey. One of the six wolves showed some evidence of its movements being influenced by prey density, but even this wolf did not select for areas of high prey density as was reported for this area (McPhee et al., 2012). The difference between studies may exist because of the analysis scale. McPhee et al. (2012) reported that at the large‐scale wolves selected hunt paths with higher prey than the overall territory, but at the scale of the hunt path landscape features rather than prey density influenced movements. In our approach, we focused on selection along the hunt path and found that the wolf selected areas of low rather than high density. In addition, we analyzed “relocating” movements and did not include short steps. Wolves possibly slow down in high prey density areas, which could have led to a removal of short steps in high prey density areas in our analysis. The movements of the wolf with an effect of prey density also showed evidence of prey management, where a predator delays a revisit to an area because a visit evokes prey behaviors that make them less vulnerable (Charnov et al., 1976; Jedrzejewski et al., 2001; Kotler, 1992; Laporte, Muhly, Pitt, Alexander, & Musiani, 2010). We had expected that wolves would revisit sites with high density sooner because there could be higher variation among individual prey relaxing post‐encounter anti‐predator behaviors and predisposing them to wolf attacks; however, when locations had been visited recently, selection by wolf w230 was highest for areas of low prey density perhaps because low densities are associated with increased vulnerability if group sizes are small (Bergmann et al., 2006; Hebblewhite & Pletscher, 2002; Kuzyk, Kneteman, & Schmiegelow, 2004).

From a modeling point of view, we were able to test the influence of time since last visit separately for territory maintenance and for foraging behaviors; however, an integration of the two behaviors within one model was more difficult. For wolf w230, the combined model fit better than the territory surveillance and the prey management model alone. But parameter estimates of the weighting function in the combined model suggested an over‐fit to spurious effects of the spatial attributes at their most extreme values. A possible explanation is that wolves make decisions in a way that our logistic weighting function was unable to represent. The logistic function could track earlier or later returns to locations based on distance to edge or prey density. However, wolves may assimilate territorial and foraging behaviors in a different nonlinear way (Rothley, Schmitz, & Cohon, 1997). We suggest further research along this line, possibly by modifying the form of weighting function in our modeling framework.

Our model discretizes both space and time, which has implications for the generality of our results. In our random‐walk model, we implicitly assume that temporal scales of the underlying behavioral process and our data (2‐hourly) match. This is a common problem when fitting discrete‐time movement models to data for statistical inference, leading to parameter estimates that are tied to the scale of the analysis and that may not necessarily agree with the “true” parameter values at the scale of the behavioral process (Schlägel & Lewis, 2016). Despite this, we believe our results qualitatively reflect the wolves’ behavior, also because we used a logistic form of the weighting function instead of an exponential form; the former having performed better in a simulation‐based analysis of the robustness of resource‐selection type movement models (Schlägel & Lewis, 2016).

In general, impact of spatial resolution is less clearly understood. In our analysis, we used a relatively coarse discretization of 300 × 300 m cells. Using a finer discretization would have increased computational burden because the bottleneck during likelihood function optimization was the computation of the normalization constant in the step probability (eqn (1)). This constant requires multiplication of kernel and weighting function for all locations within an area that the individual may possibly move to based on the current location (and this constant has to be computed for every data point in the time series). For a finer spatial resolution, the same area would consist of more locations, which would (nonlinearly) increase the amount of calculations necessary. With increasing computational power, or by further streamlining the code, it may be possible to reduce current runtime (1–2 days for our six wolves using multiple CPUs). However, we considered the discretization sufficient because of the design of TSLV in our model. For calculating TSLV, we used a buffer of about 1.2 km around the straight line between consecutive GPS fixes because wolf passage affects prey behavior beyond the actual movement path (Latombe et al., 2014; Liley & Creel, 2008). Therefore, for the sake of TSLV, a finer spatial discretization would not have increased the resolution biologically. Ideally, the size of the buffer would be integrated as a free parameter that is estimated during model fitting, in which case it could vary for different models (e.g., prey management and territory surveillance). In our analysis, we fixed the buffer size to keep model complexity at a reasonable level given the limited time series length of our data.

The approach in this paper provides a step forward in the ongoing attempt to incorporate cognition and memory in movement analyses (Avgar et al., 2015; Börger, Dalziel, & Fryxell, 2008; Fagan et al., 2013; Oliveira‐Santos, Forester, Piovezan, Tomas, & Fernandez, 2016). Our method goes beyond previous approaches that investigate traplining (Ohashi, Leslie, & Thomson, 2008) or periodicity in recursive movement patterns (Bar‐David et al., 2009; English et al., 2014; Giotto, Gerard, Ziv, Bouskila, & Bar‐David, 2015). In our models, time since last visit to locations is a spatially explicit feature that influences movement decisions in combination with information on territory geometry and prey densities. This allowed us to investigate behaviorally complex movement strategies in wolves, and we demonstrated that time since last visit influenced future movement decisions in relation to territory surveillance and prey management. Our approach can similarly be used to study the effect of time since last visit in other contexts of resource renewal (e.g., D'Souza, Patankar, Arthur, Marbà, & Alcoverro, 2015; Janmaat et al., 2006).

Despite some progress in studying cognitive aspects of animal movement, few studies have quantified the temporal and spatial scales at which individuals are aware of and respond to non‐local information. Reported time spans during which ungulates shift their habitat selection after wolf presence range from 1 day (Creel, Winnie, Maxwell, Hamlin, & Creel, 2005) to up to 10 days (Latombe et al., 2014). In contrast, Avgar et al. (2015) found indication of no memory decay in a space‐use analysis of woodland caribou. In our study, wolf w230 showed a varying response to prey density within approximately 5 days since last visit, after which the probability of selection leveled off at one for all locations. Similarly, after approximately 7 days of absence, wolf movement decisions became irrespective of distance from territory edge. These estimates are roughly in line with the scales reported by Latombe et al. (2014). In a predator–prey system where predators win the behavioral response race (Sih, 2005) we may expect predators’ response times to be larger than the prey's response, and vice versa. We need more studies that track predators and prey simultaneously and analyze the temporal scales of awareness for both predators and prey to elucidate this further. Simultaneous tracking studies have the additional advantage that temporal scales of data can be matched. As discussed above, we should expect parameter estimates from resource‐selection type analyses to be scale dependent. Unless we use truly robust models, comparisons of cognitive awareness are best to be attempted when models make the same assumptions about the scales of the behavioral processes.

In our analysis, we used a fixed buffer size for modeling the spatial extent at which locations were considered “visited” for the purpose of calculating TSLV. A possible extension of our model would treat the buffer size as a free parameter to be estimated during model fitting. With this, it would be possible to also gauge the spatial scale at which individuals experience their environment for this specific purpose.

Using information on elapsed times (“how long ago?”) can be part of episodic‐like memory in animals, a complex form of memory on the what, when, and where of events, which has been demonstrated in experiments in birds, rodents, and apes (Clayton & Dickinson, 1998; Martin‐Ordas, Haun, Colmenares, & Call, 2010; Roberts et al., 2008). Wolves may store and retrieve information on elapsed times in internal memory (Jacobs, Allen, Nguyen, & Fortin, 2013; Lew, 2011), but wolves may also use externalized memory in the form of their own scent marks (Peters & Mech, 1975), as has been argued for neurologically simple amoebae (Reid, Beekman, Latty, & Dussutour, 2013). However, whereas scent marks need to be encountered to retrieve information on previous visits, internal memory allows more efficient integration of information for goal‐oriented movement (Asensio & Brockelman, 2011; Polansky et al., 2015). Therefore, including goal‐oriented movement rules in a modeling framework such as ours would further elucidate the importance of internal memory.

## CONFLICT OF INTEREST

None declared.

## DATA ACCESSIBILITY

Data available from the Dryad Digital Repository: https://doi.org/10.5061/dryad.2j125.

Processed data and R code to run the analysis for one individual can be found online in the Supporting Information for this article.

## Figures and Tables

**Figure A1:**
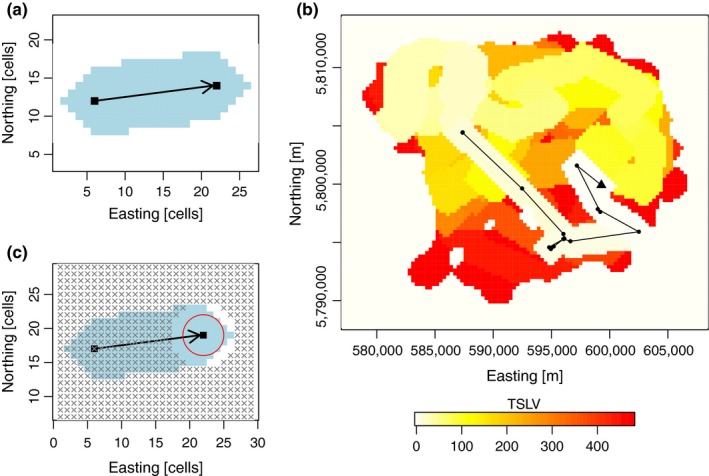
Depiction of the dynamic map TSLV. (a) TSLV is reset to 1 for locations within a buffer area (blue) around the straight‐line path (black line) between current and previous location (black cells). (b) Example for the dynamic map TSLV at one particular time step, here depicted for wolf w220. The current location is marked with a black triangle, and the last 20 time steps are depicted by black dots and black lines. The recent path of the wolf has low values of TSLV (white to light yellow), and areas with long absence have high values of TSLV (red). (c) All steps (marked by crosses) that are possible as “relocating” steps, when relocating steps are chosen to have step length ≥5 cells (~1500 m). The red circle represents locations that are within 1000 m of the current location. Steps of this length (or shorter) would naturally end at a location with TSLV = 1.

## APPENDIX A1

### COMBINED PREY DATA

To calculate the combined prey density measure, we calculated a weighted sum of prey numbers of all four species. That is, our measure of prey density was

$$N_{\mathrm{prey}}=w_{\mathrm{deer}}\times N_{\mathrm{deer}}+w_{\mathrm{elk}}\times N_{\mathrm{elk}}+w_{\mathrm{moose}}\times N_{\mathrm{moose}}+w_{\mathrm{horse}}\times N_{\mathrm{horse}}$$

where *N* is the number of individuals of the prey species indicated in the subscript, and *w* is a weight between 0 and 1 to adjust for the size of the prey. The weights were based on ungulate bodymass in winter (Knopff *et al*. 2010), for simplicity averaged over female and males,

$$b_{\mathrm{deer}}=\;82.5,\;\;b_{\mathrm{elk}}=\;275,\;\;b_{\mathrm{moose}}=\;434,\;\;b_{\mathrm{horse}}=\;420$$

To make the weights unitless, we converted them to a number between 0 and 1 by dividing the value of each species by the sum of all,

$$w_{\mathrm{species}}=\frac{b_{\mathrm{species}}}{b_{\mathrm{deer}}+b_{\mathrm{elk}}+b_{\mathrm{moose}}+b_{\mathrm{horse}}}.$$

### TIME SINCE LAST VISIT

We defined the variable *TSLV* to specify at each time step *t*, and for each location *x*, the time, measured in time steps, since the animal had last been to the location, denoted by *m*_*t*_(*x*). For example, if between times *t*−1 and *t* the animal moved from location *x*_t−1_ to *x*_*t*_, we considered all locations on the path from *x*_t−1_ to *x*_*t*_ as most recently visited and and set their value of *TSLV* at time *t* to be 1. That is, we defined *m*_*t*_(*z*) = 1 for all locations *z* that lie on the path between *x*_*t*−1_ and *x*_*t*_. For the calculation of *TSLV*, we defined the path to be the straight line between two locations. Because it is unlikely that an individual moves in a straight line, we also considered locations within a certain distance of the line as visited (for the purpose of calculating *TSLV*). For these locations, TSLV was also set to 1. Because we aimed to understand the influence of the travel history in relation to prey, we took into account at which distances wolf presence influences prey behavior. Studies on elk–wolf relationships found that wolf presence can affect elk behavior, such as group size, vigilance, and movement rates, at distances of 1–5 km (Liley & Creel 2008; Proffitt *et al*. 2009). Here, we used discretized space with landscape cells of size 300 × 300 m. We defined a buffer around a cell using the rectilinear distance measure (more colloquially also referred to as Manhattan distance)

$$d\left(x,y\right)=\left|x_{\mathrm{east}}-y_{\mathrm{north}}\right|+\left|x_{\mathrm{north}}-\;y_{\mathrm{north}}\right|,$$

where *x* and *y* are two locations on the grid with easting (*x*‐axis) and northing (*y*‐axis) coordinates *x*_east_, *y*_east_ and *x*_north_, *y*_north_, respectively. The coordinates were taken from the center of each cell. With this distance measure, the buffer becomes a diamond‐shaped area around the center cell. If we define a buffer of size δ around the location *x*, the corners of the buffer area are those cells that are δ cells away from x in exact northern, eastern, southern, and western directions. For the calculation of *TSLV*, we used a buffer of size of four cells. We calculated the buffer for each cell that is intersected by the straight line of a step (Fig. A1,a). A distance of four cells in the discretized space corresponds to 1.2 km in continuous space. Using a buffer around the straight line between two locations was a simple way of accounting for the fact that we did not observe all locations that an animal visited on its path. A more sophisticated approach would be to implement, for example, a Brownian bridge for the estimated path between two successive locations (Horne *et al*. 2007). One could even go further and expand a Brownian bridge model to include the more complex movement mechanisms studied here.

For all other locations that were not considered visited, *TSLV* increased by 1 at every time step. That is, we set *m*_*t*_(*z*) = *m*_*t*−1_(*z*) + 1 for locations *z* not visited during times *t*−1 and *t*. This led to a map with values of *TSLV* similar to a map with environmental attributes, but which changed at every time step. *TSLV* increased in areas that the individual stayed away from and was reset to 1 whenever an individual visited a location, that is, when it came sufficiently close to the location (Fig. A1,b). The dynamic map was updated at the end of each movement step, and therefore, the weighting function *w* at time *t* was based on *TSLV* at time *t*−1.

Given TSLV for some point in time, it is straightforward to update it for all following time steps based on the animal's movement path. To obtain an initial map of *TSLV*, we separated movement trajectories into two segments. We used the first 300 movement steps to initialize *TSLV* and used the rest of the trajectory for statistical inference. The time that corresponded to the beginning of the second part of the trajectory was set to be *t *= 1. We calculated *TSLV* at *t *= 1 for all locations that were visited during the initialization phase. For locations that were not visited, we set *TSLV* to the length of the initialization phase.

The trajectories contained missed observations. If at a time step *t* the corresponding location was missing, we updated *TSLV* by increasing *TSLV* for all locations by 1. We did not reset any value to 1 because there was no current path available. However, we accounted for this later at the next available time step. At that time, we reset *TSLV* to 1 for the entire path since the last available location. Because at least two time intervals had passed since the last location, we increased the buffer size for these longer steps by 2.

### MOVEMENT KERNEL

The general movement kernel *k* is the density function of a random walk in discretized two‐dimensional space. For this, we sampled a continuous‐space density at discrete points (representing the center location of each cell in the landscape). The normalization constant in the step probability given in the manuscript assures that step probabilities are properly normalized over the discretized space. We used a Weibull distribution for step lengths and assumed a uniform distribution for bearings. A major reason for using simply a uniform distribution for bearings was to retain as many steps as possible. In a correlated random walk, bearings are autocorrelated, and therefore, three successive location measurements are needed to define the probability for one movement step. With missing measurements in the time series, this would decrease the number of available steps. For example, for wolf w233 with 177 steps available for analysis, this would remove 21 of those steps, despite a reasonable fix rate of 84%. Given that using autocorrelated bearings would also add a parameter, this would reduce the ratio of available data points and number of parameters to 17 (from 22).

Thus, the kernel is given by

$$k\left(x;y\right)=\;\frac{1}{2\pi \|x-y\|}\frac{\lambda }{\sigma }{\left(\frac{\left\|x-y\right\|}{\sigma }\right)}^{\lambda -1}\exp\left(-{\left(\frac{\left\|x-y\right\|}{\sigma }\right)}^{\lambda }\right),$$

where λ and σ are the shape and scale parameter of the Weibull distribution, respectively. The factor 1/|| *x*−*y* || is due to a change from polar coordinates (using step lengths and bearings to define a step) to Euclidean spatial coordinates.

### RELOCATING STEPS

We only analyzed steps with a minimum length. Franke et al. (2006) used a hidden Markov model to identify the three major modes “bedding,” “localized activity,” and “relocating” in wolf behavior. They found that the relocating mode was characterized by steps with length above 200 m, with the majority of steps between 500 and 2500 m. These distances were obtained using movement data with hourly location measurements and therefore were not immediately transferrable to our study with 2‐hourly movement data. Roughly, steps at a rate of 500 m per hour may be converted to 1000 m per 2 hr although it is known that measurements of travel distance are influenced by sampling rate, and the longer the time interval between location measurements the larger the risk of underestimating true travel distance (Pépin *et al*. 2004; Rowcliffe *et al*. 2012). Still, with these considerations, it seemed appropriate to set the threshold for defining relocating steps at about 1000 m. If movement was straight in east‐west or north‐south direction, 1000 m corresponded to about three cells in the discretized space. Another point to consider for the threshold was the use of the buffer for *TSLV*. If a step was within the buffer size of the last visited location, the step naturally ended at a location with *TSLV *= 1 (Fig. A1,c). In contrast, if a step was larger than the buffer size, which was four cells (~1200 m), it could end at a location with *TSLV *= 1, especially when the animal backtracked. However, there was also a chance that the step ended in a location outside the buffer of the previous step with *TSLV* > 1. To avoid an artificial bias toward smaller values of *TSLV* for small steps, we defined the minimum step length to be five cells, corresponding roughly to 1,500 m in continuous space (Fig. A1,c).

Using only steps with a minimum length, strictly speaking we would have to adjust the movement kernel *k* by truncating the Weibull distribution at the minimum step length. This would lead to a slightly lower estimated mean step length for all models. Given the fairly large mean step lengths, we did not consider this problematic. In addition, we did not implement the truncated Weibull because our aim was to restrict the analysis to steps that can be attributed to a “relocating” behavioral mode. Ideally, a distinction of behavioral modes is performed by other means, for example, using a hidden Markov model (McClintock *et al*. 2012), in which case “relocating” steps could have also smaller step length. We did not embed our model into a hidden Markov model, because based on our restricted time series length, we could not increase model complexity arbitrarily. However, for data sets with longer time series length, for example, due to higher temporal resolution, we recommend more sophisticated approaches to data segmentation.

### LIKELIHOOD FUNCTION AND OPTIMIZATION

The likelihood function of the model is

$$L\left(\lambda ,\sigma ,\alpha ,\beta ,\gamma \right)=\;\prod_{i}^{N}p(x_{t_{i}}\;|x_{t_{i-1}},\;\lambda ,\sigma ,\alpha ,\beta ,\gamma )$$

for all available steps from $x_{t_{i-1}}$ to $x_{t_{i}}$ with $\;\|x_{t_{i-1}}-x_{t_{i}}\|\;\geq 5$. During model fitting, we conditioned the likelihood on the first location of each segment of successively available locations.

Note that nonrelocating steps were omitted after calculating *TSLV* for the entire time series. Therefore, steps used for the final analysis had appropriate values of *TSLV*, representing correct times based on the full path.

We optimized the likelihood function using a Nelder–Mead algorithm implemented in R (R Core Team 2015). To find the find global maximum, we optimized the likelihood function starting at various points in parameter space. From these results, we chose the parameter with the highest likelihood value and used them as starting point for the final optimization. We used an estimate of the Hessian matrix of the log‐likelihood at the optimal parameter values to obtain standard errors of the maximum‐likelihood estimates.

## APPENDIX A2

### A2.2

**Table A1:** Model selection results when the models with time‐dependent effect for both distance from edge and prey density (last two rows) were included. Presented are AIC_c_ differences, ΔAIC_c,i_ = AIC_c,i_ − AIC_c,min_ for each model i. Best models are highlighted in bold. For individual w230 the most complex model becomes best, however parameter estimates suggest that the model is an over‐fit to spurious effects of extreme values of the spatial attributes (Table A2, Fig. A2)

	ΔAIC_c_					
	w83	w220	w230	w233	w284	w285
null	59.7	66.4	60.3	63.4	126.3	58.0
edge	45.2	66.6	58.0	49.7	77.0	38.2
TSLV+edge	17.6	6.5	10.3	27.7	53.8	23.6
**TSLV+edge+TSLV*edge**	**0**	**0**	2.4	**0**	**0.4**	**0**
prey	63.0	60.7	60.2	67.3	130.4	59.0
edge+prey	45.6	67.7	47.1	43.3	72.9	33.1
edge+prey+edge*prey	47.7	69.6	45.9	41.6	74.7	35.1
TSLV	16.1	5.8	8.2	36.3	52.2	22.7
TSLV+prey	18.0	5.9	2.4	30.2	53.1	23.2
TSLV+prey+TSLV*prey	18.0	6.0	4.5	32.0	54.1	22.7
TSLV+edge+prey	19.6	7.3	4.4	29.1	54.0	24.1
**TSLV+edge+prey+TSLV*edge** ***+*** **TSLV** ******* **prey**	2.1	2.1	**0**	1.9	0	0.9

**Table A2:** Parameter estimates for the model with interaction of both distance from edge and prey density with TSLV. Standard errors for the selection and interaction coefficients are all larger than the estimates themselves, leading to large Wald‐type confidence intervals that overlap zero, indicating high uncertainty in the estimates

	α	β_tslv_	β_edge_	β_prey_	γ_edge_	γ_prey_
w230						
Est.	−3.57	2.49	−0.07	−1.3	1.64	3.43
SE	1.73	2.93	0.13	1.56	2.44	5.22

## APPENDIX A3

### Runtime benchmarks

Here, we provide an overview of the computational load of the model fitting. The measurements provided here result from test runs on a PC with Intel Core i5 2.3 GHz processor. We used data of wolf 220. Model fitting required optimization of the likelihood function over a multidimensional parameter space. For the null model, there were only two parameters to estimate, while the most complex model contained eight parameters (those presented in Table A2, plus *σ* and *λ*). One evaluation of the likelihood function required approximately 0.54 seconds. Most of this runtime can be attributed to computation of the normalization constant (denominator) in eqn 1. During the optimization routine, the likelihood function has to be evaluated many times. The amount of calls to the likelihood function necessary until the optimization routing converges varies depending on which model is fitted. During our test runs, the null model required up to 93 calls to the function; the territory surveillance model (H_2b_) required up to 1147 calls; the most complex model required up to 2000 calls (which we had set as maximum number of iterations in the optim function in R). To find a global maximum in multidimensional parameter space, it is customary to perform the optimization multiple times with varying starting values. We used 20 starting values and used the resulting optimum for the final optimization. Thus, if we use 1000 iterations until convergence as a baseline, one model fit requires approximately 190 minutes. We had 10 models (Table 1), not counting the additional runs with the most complex model (Table A1), and six wolves, resulting in an approximate runtime of 8 days. Note that the actual runtime, however, can be shortened by running the analysis simultaneously on multiple CPUs.

## References

[ece33176-bib-0001] Aarts, G. , Fieberg, J. , & Matthiopoulos, J. (2012). Comparative interpretation of count, presence‐absence and point methods for species distribution models. Methods in Ecology and Evolution, 3, 177–187.

[ece33176-bib-0002] Asensio, N. , & Brockelman, W. Y. (2011). Gibbon travel paths are goal oriented. Animal Cognition, 14, 395–405. 21221693 10.1007/s10071-010-0374-1

[ece33176-bib-0003] Ausband, D. E. , Mitchell, M. S. , Bassing, S. B. , & White, C. (2013). No trespassing: Using a biofence to manipulate wolf movements. Wildlife Research, 40, 207–216.

[ece33176-bib-0004] Avgar, T. , Baker, J. A. , Brown, G. S. , Hagens, J. S. , Kittle, A. M. , Mallon, E. E. , … Fryxell, J. M. (2015). Space‐use behaviour of woodland caribou based on a cognitive movement model. Journal of Animal Ecology, 84, 1059–1070. 25714592 10.1111/1365-2656.12357

[ece33176-bib-0005] Avgar, T. , Deardon, R. , & Fryxell, J. M. (2013). An empirically parameterized individual based model of animal movement, perception, and memory. Ecological Modelling, 251, 158–172.

[ece33176-bib-0006] Avgar, T. , Potts, J. R. , Lewis, M. A. , & Boyce, M. S. (2016). Integrated step selection analysis: Bridging the gap between resource selection and animal movement. Methods in Ecology and Evolution, 7, 619–630.

[ece33176-bib-0007] Bar‐David, S. , Bar‐David, I. , Cross, P. C. , Ryan, S. J. , Knechtel, C. U. , & Getz, W. M. (2009). Methods for assessing movement path recusrion with application to African buffalo in South Africa. Ecology, 90, 2467–2479. 19769125 10.1890/08-1532.1 PMC3025599

[ece33176-bib-0008] Basille, M. , Fortin, D. , Dussault, C. , Bastille‐Rousseau, G. , Ouellet, J. P. , & Courtois, R. (2015). Plastic response of fearful prey to the spatiotemporal dynamics of predator distribution. Ecology, 96, 2622–2631. 26649384 10.1890/14-1706.1

[ece33176-bib-0009] Berger‐Tal, O. , & Bar‐David, S. (2015). Recursive movement patterns: Review and synthesis across species. Ecosphere, 6, art149.

[ece33176-bib-0010] Bergmann, E. J. , Garrott, R. A. , Creel, S. , Borkowski, J. J. , Jaffe, R. , & Watson, F. G. R. (2006). Assessment of prey vulnarability through analysis of wolf movements and kill sites. Ecological Applications, 16, 273–284. 16705979 10.1890/04-1532

[ece33176-bib-0011] Börger, L. , Dalziel, B. D. , & Fryxell, J. M. (2008). Are there general mechanisms of animal home range behaviour? A review and prospects for future research. Ecology Letters, 11, 637–650. 18400017 10.1111/j.1461-0248.2008.01182.x

[ece33176-bib-0012] Bracis, C. , Gurarie, E. , Van Moorter, B. , & Goodwin, R. A. (2015). Memory effects on movement behavior in animal foraging. PLoS ONE, 10, 1–21. 10.1371/journal.pone.0136057 PMC4542208 26288228

[ece33176-bib-0013] Brown, J. S. , Laundré, J. W. , & Gurung, M. (1999). The ecology of fear: Optimal foraging, game theory, and trophic interactions. Journal of Mammalogy, 80, 385–399.

[ece33176-bib-0014] Burnham, K. P. , & Anderson, D. R. (2002). Model selection and multimodel inference: A practical information‐theoretic approach, 2nd ed New York, NY: Springer.

[ece33176-bib-0015] Burns, J. G. , Foucaud, J. , & Mery, F. (2011). Costs of memory: Lessons from “mini” brains. Proceedings of the Royal Society B: Biological Sciences, 278, 923–929. 21177679 10.1098/rspb.2010.2488 PMC3049060

[ece33176-bib-0500] Calenge, C. (2006). The package “adehabitat” for the R software: A tool for the analysis of space and habitat use by animals. Ecological Modelling, 197, 516–519.

[ece33176-bib-0016] Carbyn, L. N. (1983). Wolf predation on elk in Riding Mountain National Park, Manitoba. The Journal of Wildlife Management, 47, 963–976.

[ece33176-bib-0017] Charnov, E. L. , Orians, G. H. , & Hyatt, K. (1976). Ecologial implications of resource depression. The American Naturalist, 110, 247–259.

[ece33176-bib-0018] Clayton, N. S. , & Dickinson, A. (1998). Episodic‐like memory during cache recovery by scrub jays. Nature, 395, 272–274. 9751053 10.1038/26216

[ece33176-bib-0019] Creel, S. , Winnie, J. A. , Christianson, D. , & Liley, S. (2008). Time and space in general models of antipredator response: Tests with wolves and elk. Animal Behaviour, 76, 1139–1146.

[ece33176-bib-0020] Creel, S. , Winnie, J. , Maxwell, B. , Hamlin, K. , & Creel, M. (2005). Elk alter habitat selection as an antipredator response to wolves. Ecology, 86, 3387–3397.

[ece33176-bib-0021] Davies, N. B. , & Houston, A. I. (1981). Owners and satellites: The economics of territory defence in the pied wagtail, *Motacilla alba* . Journal of Animal Ecology, 50, 157–180.

[ece33176-bib-0022] D'Souza, E. , Patankar, V. , Arthur, R. , Marbà, N. , & Alcoverro, T. (2015). Seagrass herbivory levels sustain site‐fidelity in a remnant dugong population. PLoS ONE, 10, 1–18. 10.1371/journal.pone.0141224 PMC4619644 26492558

[ece33176-bib-0023] English, M. , Ancrenaz, M. , Gillespie, G. , Goossens, B. , Nathan, S. , & Linklater, W. (2014). Foraging site recursion by forest Elephants *Elephas maximus borneensis* . Current Zoology, 60, 551–559.

[ece33176-bib-0024] Fagan, W. F. , Lewis, M. A. , Auger‐Méthé, M. , Avgar, T. , Benhamou, S. , Breed, G. , … Mueller, T. (2013). Spatial memory and animal movement. Ecology Letters, 16, 1316–1329. 23953128 10.1111/ele.12165

[ece33176-bib-0025] Feldhamer, G. A. , Drickamer, L. C. , Vessey, S. H. , & Merritt, J. F. (2004). Mammalogy: Adaptation, diversity, ecology, 2nd ed New York, NY: McGraw‐Hill.

[ece33176-bib-0026] Forester, J. D. , Im, H. K. , & Rathouz, P. J. (2009). Accounting for animal movement in estimation of resource selection functions: Sampling and data analysis. Ecology, 90, 3554–3565. 20120822 10.1890/08-0874.1

[ece33176-bib-0027] Fortin, D. , Beyer, H. L. , Boyce, M. S. , Smith, D. W. , Duchesne, T. , & Mao, J. S. (2005). Wolves influence elk movements: Behavior shapes a trophic cascade in Yellowstone National Park. Ecology, 86, 1320–1330.

[ece33176-bib-0028] Frair, J. L. , Nielsen, S. E. , Merrill, E. H. , Lele, S. R. , Boyce, M. S. , Munro, R. H. M. , … Beyer, H. L. (2004). Removing GPS collar bias in habitat selection studies. Journal of Applied Ecology, 41, 201–212.

[ece33176-bib-0029] Franke, A. , Caelli, T. , Kuzyk, G. , & Hudson, R. J. (2006). Prediction of wolf (*Canis lupus*) kill‐sites using hidden Markov models. Ecological Modelling, 197, 237–246.

[ece33176-bib-0030] Giotto, N. , Gerard, J. F. , Ziv, A. , Bouskila, A. , & Bar‐David, S. (2015). Space‐use patterns of the Asiatic wild ass (*Equus hemionus*): Complementary insights from displacement, recursion movement and habitat selection analyses. PLoS ONE, 10, 1–21. 10.1371/journal.pone.0143279 PMC4667895 26630393

[ece33176-bib-0031] Grove, M. (2013). The evolution of spatial memory. Mathematical Biosciences, 242, 25–32. 23246804 10.1016/j.mbs.2012.11.011

[ece33176-bib-0032] Hebblewhite, M. , Percy, M. , & Merrill, E. H. (2007). Are all global positioning system collars created equal? Correcting habitat‐induced bias using three brands in the Central Canadian Rockies. Journal of Wildlife Management, 71, 2026–2033.

[ece33176-bib-0033] Hebblewhite, M. , & Pletscher, D. H. (2002). Effects of elk group size on predation by wolves. Canadian Journal of Zoology, 80, 800–809.

[ece33176-bib-0034] Holling, C. S. (1959). The components of predation as revealed by a study of small‐mammal predation of the European pine sawfly. The Canadian Entomologist, 91, 293–320.

[ece33176-bib-0035] Hopkins, M. E. (2015). Mantled howler monkey spatial foraging decisions reflect spatial and temporal knowledge of resource distributions. Animal Cognition, 19, 1–17. 26597923 10.1007/s10071-015-0941-6

[ece33176-bib-0036] Horne, J. S. , Garton, E. O. , Krone, S. M. , & Lewis, J. S. (2007). Analyzing animal movements using Brownian bridges. Ecology, 88, 2354–2363. 17918412 10.1890/06-0957.1

[ece33176-bib-0037] Jacobs, N. S. , Allen, T. A. , Nguyen, N. , & Fortin, N. J. (2013). Critical role of the hippocampus in memory for elapsed time. The Journal of Neuroscience, 33, 13888–13893. 23966708 10.1523/JNEUROSCI.1733-13.2013 PMC6618651

[ece33176-bib-0038] Janmaat, K. R. L. , Byrne, R. W. , & Zuberbühler, K. (2006). Evidence for a spatial memory of fruiting states of rainforest trees in wild mangabeys. Animal Behaviour, 72, 797–807.

[ece33176-bib-0039] Jedrzejewski, W. , Schmidt, K. , Theuerkauf, J. , Jedrzejewska, B. , & Okarma, H. (2001). Daily movements and territory use by radio‐collared wolves (*Canis lupus*) in Bialowieza Primeval Forest in Poland. Canadian Journal of Zoology, 79, 1993–2004.

[ece33176-bib-0040] Knopff, K. H. , Knopff, A. A. , Kortello, A. , & Boyce, M. S. (2010). Cougar kill rate and prey composition in a multiprey system. Journal of Wildlife Management, 74, 1435–1447.

[ece33176-bib-0041] Kotler, B. P. (1992). Behavioral resource depression and decaying perceived risk of predation in two species of coexisting gerbils. Behavioral Ecology and Sociobiology, 30, 239–244.

[ece33176-bib-0042] Kuijper, D. P. J. , Verwijmeren, M. , Churski, M. , Zbyryt, A. , Schmidt, K. , Jedrzejewska, B. , & Smit, C. (2014). What cues do ungulates use to assess predation risk in dense temperate forests? PLoS ONE, 9, 1–12. 10.1371/journal.pone.0084607 PMC3880296 24404177

[ece33176-bib-0043] Kunkel, K. , & Pletscher, D. H. (2001). Winter hunting patterns of wolves in and near Glacier National Park, Montana. The Journal of Wildlife Management, 65, 520–530.

[ece33176-bib-0044] Kuzyk, G. W. , Kneteman, J. , & Schmiegelow, F. K. A. (2004). Winter habitat use by wolves, Canis lupus, in relation to forest harvesting in West‐central Alberta. The Canadian Field‐Naturalist, 118, 368–375.

[ece33176-bib-0045] Laporte, I. , Muhly, T. B. , Pitt, J. A. , Alexander, M. , & Musiani, M. (2010). Effects of wolves on elk and cattle behaviors: Implications for livestock production and wolf conservation. PLoS ONE, 5, e11954. 20694139 10.1371/journal.pone.0011954 PMC2915913

[ece33176-bib-0046] Latombe, G. , Fortin, D. , & Parrott, L. (2014). Spatio‐temporal dynamics in the response of woodland caribou and moose to the passage of grey wolf. Journal of Animal Ecology, 83, 185–198. 23859231 10.1111/1365-2656.12108

[ece33176-bib-0047] Laundré, J. W. (2010). Behavioral response races, predator‐prey shell games, ecology of fear, and patch use of pumas and their ungulate prey. Ecology, 91, 2995–3007. 21058559 10.1890/08-2345.1

[ece33176-bib-0048] Lele, S. R. , Merrill, E. H. , Keim, J. , & Boyce, M. S. (2013). Selection, use, choice and occupancy: Clarifying concepts in resource selection studies. Journal of Animal Ecology, 82, 1183–1191. 24499379 10.1111/1365-2656.12141

[ece33176-bib-0049] Lew, A. R. (2011). Looking beyond the boundaries: Time to put landmarks back on the cognitive map? Psychological Bulletin, 137, 484–507. 21299273 10.1037/a0022315

[ece33176-bib-0050] Lewis, M. A. , & Murray, J. D. (1993). Modelling territoriality and wolf‐deer interactions. Nature, 366, 738–740.

[ece33176-bib-0051] Liley, S. , & Creel, S. (2008). What best explains vigilance in elk: Characteristics of prey, predators, or the environment? Behavioral Ecology, 19, 245–254.

[ece33176-bib-0052] Lima, S. L. (2002). Putting predators back into behavioral predator–prey interactions. Trends in Ecology & Evolution, 17, 70–75.

[ece33176-bib-0053] Martin‐Ordas, G. , Haun, D. , Colmenares, F. , & Call, J. (2010). Keeping track of time: Evidence for episodic‐like memory in great apes. Animal Cognition, 13, 331–340. 19784852 10.1007/s10071-009-0282-4 PMC2822233

[ece33176-bib-0055] McPhee, H. M. , Webb, N. F. , & Merrill, E. H. (2012). Hierarchial predation: Wolf (*Canis lupus*) selection along hunt paths and at kill sites. Canadian Journal of Zoology, 90, 555–563.

[ece33176-bib-0056] Mech, L. D. (1994). Buffer zones of territories of gray wolves as regions of intraspecific strife. Journal of Mammology, 75, 199–202.

[ece33176-bib-0057] Mech, L. D. , & Boitani, L. (Eds.) (2006). Wolves: Behaviour, ecology, and conservation. Chicago, IL: University of Chicago Press.

[ece33176-bib-0058] Mech, L. D. , & Harper, E. K. (2002). Differential use of a wolf, *Canis lupus*, pack territory edge and core. The Canadian Field‐Naturalist, 116, 315–316.

[ece33176-bib-0059] Merkle, J. A. , Fortin, D. , & Morales, J. M. (2014). A memory‐based foraging tactic reveals an adaptive mechanism for restricted space use. Ecology Letters, 17, 924–931. 24811575 10.1111/ele.12294

[ece33176-bib-0060] Merrill, E. , Sand, H. , Zimmermann, B. , McPhee, H. , Webb, N. , Hebblewhite, M. , … Frair, J. L. (2010). Building a mechanistic understanding of predation with GPS‐based movement data. Philosophical transactions of the Royal Society B, Biological sciences, 365, 2279–2288. 10.1098/rstb.2010.0077 PMC2894956 20566504

[ece33176-bib-0061] Metz, M. C. , Vucetich, J. A. , Smith, D. W. , Stahler, D. R. , & Peterson, R. O. (2011). Effect of sociality and season on gray wolf (*Canis lupus*) foraging behavior: Implications for estimating summer kill rate. PLoS ONE, 6, 1–10. 10.1371/journal.pone.0017332 PMC3046980 21390256

[ece33176-bib-0062] Mueller, T. , Fagan, W. F. , & Grimm, V. (2011). Integrating individual search and navigation behaviors in mechanistic movement models. Theoretical Ecology, 4, 341–355.

[ece33176-bib-0063] Nabe‐Nielsen, J. , Tougaard, J. , Teilmann, J. , Lucke, K. , & Forchhammer, M. C. (2013). How a simple adaptive foraging strategy can lead to emergent home ranges and increased food intake. Oikos, 122, 1307–1316.

[ece33176-bib-0064] Ohashi, K. , Leslie, A. , & Thomson, J. D. (2008). Trapline foraging by bumble bees: V. Effects of experience and priority on competitive performance. Behavioral Ecology, 19, 936–948.

[ece33176-bib-0065] Oliveira‐Santos, L. G. R. , Forester, J. D. , Piovezan, U. , Tomas, W. M. , & Fernandez, F. A. S. (2016). Incorporating animal spatial memory in step selection functions. Journal of Animal Ecology, 85, 516–524. 26714244 10.1111/1365-2656.12485

[ece33176-bib-0067] Peters, R. P. , & Mech, L. D. (1975). Scent‐marking in wolves. American Scientist, 63, 628–637. 1200478

[ece33176-bib-0068] Peterson, R. O. (1974). Wolf ecology and prey relationships on Isle Royale. United States National Park Service Scientific Monograph Series, 11, 1–210.

[ece33176-bib-0069] Polansky, L. , Kilian, W. , & Wittemyer, G. (2015). Elucidating the significance of spatial memory on movement decisions by African savannah elephants using state‐space models. Proceedings of the Royal Society B: Biological Sciences, 282, 20143042–20143042. 25808888 10.1098/rspb.2014.3042 PMC4389615

[ece33176-bib-0071] R Core Team . (2015). R: A language and environment for statistical computing. R Foundation for Statistical Computing, Vienna, Austria URL https://www.R-project.org/.

[ece33176-bib-0072] Reid, C. R. , Beekman, M. , Latty, T. , & Dussutour, A. (2013). Amoeboid organism uses extracellular secretions to make smart foraging decisions. Behavioral Ecology, 24, 812–818.

[ece33176-bib-0073] Riotte‐Lambert, L. , Benhamou, S. , & Chamaillé‐Jammes, S. (2015). How memory‐based movement leads to nonterritorial spatial segregation. The American Naturalist, 185, E103–E116. 10.1086/680009 25811090

[ece33176-bib-0074] Roberts, W. A. , Feeney, M. C. , Macpherson, K. , Petter, M. , Mcmillan, N. , & Musolino, E. (2008). Episodic‐like memory in rats: Is it based on when or how long ago? Science, 320, 113–115. 18388296 10.1126/science.1152709

[ece33176-bib-0075] Robinson, B. G. , & Merrill, E. H. (2013). Foraging‐vigilance trade‐offs in a partially migratory population: Comparing migrants and residents on a sympatric range. Animal Behaviour, 85, 849–856.

[ece33176-bib-0076] Rothley, K. D. , Schmitz, O. J. , & Cohon, J. L. (1997). Foraging to balance conflicting demands: Novel insights from grasshoppers under predation risk. Behavioral Ecology, 8, 551–559.

[ece33176-bib-0077] Rowcliffe, J. M. , Carbone, C. , Kays, R. , Kranstauber, B. , & Jansen, P. A. (2012). Bias in estimating animal travel distance: The effect of sampling frequency. Methods in Ecology and Evolution, 3, 653–662.

[ece33176-bib-0078] Schlägel, U. E. , & Lewis, M. A. (2014). Detecting effects of spatial memory and dynamic information on animal movement decisions. Methods in Ecology and Evolution, 5, 1236–1246.

[ece33176-bib-0079] Schlägel, U. E. , & Lewis, M. A. (2016). Robustness of movement models: Can models bridge the gap between temporal scales of data sets and behavioural processes? Journal of Mathematical Biology, 73, 1691–1726. 27098937 10.1007/s00285-016-1005-5

[ece33176-bib-0080] Sih, A. (2005). Predator‐prey space use as an emergent outcome of a behavioral response race. Ecology of Predator‐Prey Interactions, 240–255.

[ece33176-bib-0081] Van Moorter, B. , Visscher, D. , Benhamou, S. , Börger, L. , Boyce, M. S. , & Gaillard, J. M. (2009). Memory keeps you at home: A mechanistic model for home range emergence. Oikos, 118, 641–652.

[ece33176-bib-0082] Webb, N. , Hebblewhite, M. , & Merrill, E. (2008). Statistical methods for identifying wolf kill sites using global positioning system locations. Journal of Wildlife Management, 72, 798–807.

[ece33176-bib-0083] Zub, K. , Theuerkauf, J. , Jedrzejewski, W. J. , Jedrzejewska, B. J. , Schmidt, K. , & Kowalczyk, R. (2003). Wolf pack territory marking in the Bialowieza Primeval Forest (Poland). Behaviour, 140, 635–648.

