# Supplementary material for: Territory surveillance and prey management: Wolves keep track of space and time
Source: Ecol Evol. 2017 Sep 9;7(20):8388–405. doi: 10.1002/ece3.3176 (PMC5648667; doi:10.1002/ece3.3176)
Supplement: Supplementary file 4 [file ECE3-7-8388-s004.docx]

Table A1. Model selection results when the models with time-dependent effect for both distance from edge and prey density (last two rows) was included. Presented are AIC_c_ differences, ΔAIC_c,i_ = AIC_c,i_ – AIC_min_ for each model i. Best models are highlighted in bold. For individual w230 the most complex model becomes best, however parameter estimates suggest that the model is an over-fit to spurious effects of extreme values of the spatial attributes (Table A2, Fig. A3).

|  | **ΔAIC_c_** | | | | | |
| --- | --- | --- | --- | --- | --- | --- |
|  | **w83** | **w220** | **w230** | **w233** | **w284** | **w285** |
| null | 59.7 | 66.4 | 60.3 | 63.4 | 126.3 | 58.0 |
| edge | 45.2 | 66.6 | 58.0 | 49.7 | 77.0 | 38.2 |
| TSLV+edge | 17.6 | 6.5 | 10.3 | 27.7 | 53.8 | 23.6 |
| **TSLV+edge+TSLV*edge** | **0** | **0** | 2.4 | **0** | **0.4** | **0** |
| prey | 63.0 | 60.7 | 60.2 | 67.3 | 130.4 | 59.0 |
| edge+prey | 45.6 | 67.7 | 47.1 | 43.3 | 72.9 | 33.1 |
| edge+prey+edge*prey | 47.7 | 69.6 | 45.9 | 41.6 | 74.7 | 35.1 |
| TSLV | 16.1 | 5.8 | 8.2 | 36.3 | 52.2 | 22.7 |
| TSLV+prey | 18.0 | 5.9 | 2.4 | 30.2 | 53.1 | 23.2 |
| TSLV+prey+TSLV*prey | 18.0 | 6.0 | 4.5 | 32.0 | 54.1 | 22.7 |
| TSLV+edge+prey | 19.6 | 7.3 | 4.4 | 29.1 | 54.0 | 24.1 |
| **TSLV+edge+prey+TSLV*edge*+*TSLV***prey** | 2.1 | 2.1 | **0** | 1.9 | 0 | 0.9 |
