# Supplementary material for: Territory surveillance and prey management: Wolves keep track of space and time
Source: Ecol Evol. 2017 Sep 9;7(20):8388–405. doi: 10.1002/ece3.3176 (PMC5648667; doi:10.1002/ece3.3176)
Supplement: Supplementary file 5 [file ECE3-7-8388-s005.docx]

**Table A2.** Parameter estimates for the model with interaction of both distance from edge and prey density with TSLV. Standard errors for the selection and interaction coefficients are all larger than the estimates themselves, leading to large Wald-type confidence intervals that overlap zero, indicating high uncertainty in the estimates.

|  | **α** | **β_tslv_** | **β_edge_** | **β_prey_** | **γ_edge_** | **γ_prey_** |
| --- | --- | --- | --- | --- | --- | --- |
| **w230** | | | | | | |
| Est. | -3.57 | 2.49 | -0.07 | -1.3 | 1.64 | 3.43 |
| SE | 1.73 | 2.93 | 0.13 | 1.56 | 2.44 | 5.22 |
